# SNP identification and marker assay development for high-throughput selection of soybean cyst nematode resistance

**DOI:** 10.1186/s12864-015-1531-3

**Published:** 2015-04-18

**Authors:** Zi Shi, Shiming Liu, James Noe, Prakash Arelli, Khalid Meksem, Zenglu Li

**Affiliations:** Center for Applied Genetic Technologies & Dep. of Crop and Soil Sciences, University of Georgia, Athens, GA 30602 USA; Department of Plant, Soil and Agriculture Systems, Southern Illinois University, Carbondale, IL 62901 USA; Department of Plant Pathology, University of Georgia, Athens, GA 30602 USA; USDA-ARS-SEA, Jackson, TN 38301 USA

**Keywords:** Soybean cyst nematode, Resistance, SNP, KASP marker assays, Marker assisted selection

## Abstract

**Background:**

Soybean cyst nematode (SCN) is the most economically devastating pathogen of soybean. Two resistance loci, *Rhg1* and *Rhg4* primarily contribute resistance to SCN race 3 in soybean. Peking and PI 88788 are the two major sources of SCN resistance with Peking requiring both *Rhg1* and *Rhg4* alleles and PI 88788 only the *Rhg1* allele. Although simple sequence repeat (SSR) markers have been reported for both loci, they are linked markers and limited to be applied in breeding programs due to accuracy, throughput and cost of detection methods. The objectives of this study were to develop robust functional marker assays for high-throughput selection of SCN resistance and to differentiate the sources of resistance.

**Results:**

Based on the genomic DNA sequences of 27 soybean lines with known SCN phenotypes, we have developed Kompetitive Allele Specific PCR (KASP) assays for two Single nucleotide polymorphisms (SNPs) from Glyma08g11490 for the selection of the *Rhg4* resistance allele. Moreover, the genomic DNA of Glyma18g02590 at the *Rhg1* locus from 11 soybean lines and cDNA of Forrest, Essex, Williams 82 and PI 88788 were fully sequenced. Pairwise sequence alignment revealed seven SNPs/insertion/deletions (InDels), five in the 6th exon and two in the last exon. Using the same 27 soybean lines, we identified one SNP that can be used to select the *Rhg1* resistance allele and another SNP that can be employed to differentiate Peking and PI 88788-type resistance. These SNP markers have been validated and a strong correlation was observed between the SNP genotypes and reactions to SCN race 3 using a panel of 153 soybean lines, as well as a bi-parental population, F_5_–derived recombinant inbred lines (RILs) from G00-3213 x LG04-6000.

**Conclusions:**

Three functional SNP markers (two for *Rhg1* locus and one for *Rhg4* locus) were identified that could provide genotype information for the selection of SCN resistance and differentiate Peking from PI 88788 source for most germplasm lines. The robust KASP SNP marker assays were developed. In most contexts, use of one or two of these markers is sufficient for high-throughput marker-assisted selection of plants that will exhibit SCN resistance.

**Electronic supplementary material:**

The online version of this article (doi:10.1186/s12864-015-1531-3) contains supplementary material, which is available to authorized users.

## Background

Soybean cyst nematode (SCN, *Heterodera glycines* L.) is the most economically devastating pathogen of soybean and causes losses in soybean producing areas worldwide with no practical means of eradication [1]. The annual losses exceed one billion dollars in the United States alone [2]. Two resistance genes for SCN, *Rhg1* (Resistance to *H. glycines*) on Chromosome (Chr) 18 and *Rhg4* on Chr08, have been identified in the soybean germplasm, and have been shown to be the key sources of resistance in soybean cultivars [3]. The *Rhg1* locus, which is crucial for broad spectrum resistance to SCN, has been identified in various resistance sources, such as PI 88788 and Peking [4,5].

SCN field populations are genetically variable [6,7]. Sixteen SCN races have been described and the race designation was determined by comparing its reproduction on a set of four soybean differentials with a standard SCN-susceptible cultivar. To avoid the implication of genetic predictability, a revised classification scheme for field SCN population introduced the *Heterodera glycines* (HG) types determined by reactions to seven indicator lines [7]. The distribution of SCN populations in the field varied among different geographic regions. However, race 3 is the most frequently found SCN populations in major soybean producing states in the USA [8,9]. Soybean lines PI 88788 and Peking are the two main sources of resistance to SCN race 3 [10,11]. Over 90% of the commercially available SCN-resistant soybean cultivars in the USA carry the *rhg1-b* allele, which is derived from PI 88788 due to its desirable agronomic characteristics [1,12,13]. While PI 88788-type resistance only requires the *Rhg1* resistance allele, Peking-type resistance needs both *Rhg1* and *Rhg4* resistance alleles for a resistant phenotype [14,15]. Although the molecular basis of SCN-resistance is still unknown, recent studies have shed light on the genes involved in the resistance. Map-based cloning showed that a single gene at *Rhg4* locus encoding a serine hydroxymethyltransferase (SHMT, Glyma08g11490) is responsible for SCN resistance [14]. Two polymorphisms have been identified in the coding sequence of SHMT, 389 G/C in the first exon and 1,165 A/T in the second exon, which resulted in the amino acid change of Arginine (R) to Proline (P) and Tyrosine(Y) to Asparagine (N), respectively. These polymorphisms change the property of the enzyme and may correlate with *Rhg4*-dependent resistance and susceptibility [14]. Genetic mapping and gene functional analysis of the *rhg1-b* locus exhibited a 31.2 kb genomic segment encoding a predicted amino acid transporter (Glyma18g02580), an α–SNAP protein (Glyma18g02590) and a protein with wound-inducible protein 12 (WI12) (Glyma18g02610) [12,16]. Gene silencing and overexpression analysis showed that all three genes contribute to SCN resistance [12]. Interestingly, the phenotypic reaction to SCN is associated with the copy number variation of this 31.2 kb fragment. Only one copy is present in susceptible varieties, such as Williams 82, and multiple copies in SCN resistant lines. For example, Peking possesses three copies and seven to 10 copies were detected in PI 88788-derived resistant lines [12]. Sequencing of cDNA from several soybean lines revealed that multiple alleles of Glyma18g02590 were present in the different multi-copy *Rhg1* categories [16].

Planting of resistant soybean cultivars combined with crop rotation formulates the best management strategy for reducing yield losses due to SCN. However, traditional breeding for resistant lines is challenging, not just because the phenotyping of SCN is time-consuming, but it is also affected by the different resistance genes [17] and varied SCN populations in the field [6,7]. Therefore, the use of molecular markers to assist the selection of SCN resistance is a crucial improvement in breeding programs. Major efforts have been made to develop genetic markers linked to SCN resistance genes. Restriction fragment length polymorphisms (RFLPs) markers have been identified and reported to facilitate soybean breeding for both *Rhg1* [3,18-20] and *Rhg4* loci [15,21-23]. Simple sequence repeats (SSRs) have further been discovered and applied in the marker-assisted selection of SCN resistance [15,21,22,24-27]. SNP/InDel markers have also been developed at the flanking region of both loci [11,28-30]. However, they are not diagnostic markers, and both SSRs and RFLPs are labor/time-consuming and cost ineffective. Thus, they are very limited to be applied in breeding programs in a high throughput setting.

Here, we report the development of three robust, functional SNP marker assays, two for the *Rhg1* locus and one for *Rhg4*. These functional SNP markers provide genotype information which can be used for the high-throughput selection of SCN resistance, as well as differentiating Peking and PI 88788 resistance sources for breeding of SCN resistance cultivars and germplasm introgression.

## Results

### SNP discovery

**Figure 1:**
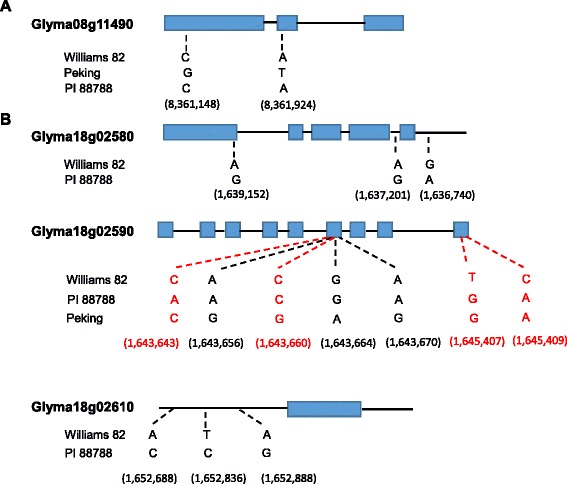
Schematic graph showed the position of SNP/InDels at *Rhg1* and *Rhg4* loci. **A**. Two SNPs identified at Glyma08g11490 at *Rhg4* locus. **B**. 13 SNP/InDels identified in three genes within a 31.2 kb block at *Rhg1* locus. Blue squares represent exons and black lines represent UTRs and/or introns. The genome coordinates (Database: Gmax_275_v2.0.softmasked) are shown under each SNP in parenthesis. SNP alleles in black are from published sequences and SNP alleles in red from our sequencing results.

To determine SNP markers within the *Rhg1* locus, published sequences of the region were gathered which included the entire 31.2 kb block from PI 88788 and Williams 82 [12], but only the cDNA of Glyma18g02590 from Peking was available [31]. Moreover, we fully sequenced the genomic DNA of Glyma18g02590 from 11 soybean lines and cDNA of Glyma18g02590 from Forrest, Essex, Williams 82 and PI 88788 at the *Rhg1* locus. Sequence alignment was only carried out for Glyma18g02580, Glyma18g02590 and Glyma18g02610, because other genes (partial Glyma18g02570 and Glyma18g02600) in this 31.2 kb block are not functional according to the previous study [12]. Three SNPs were identified in Glyma18g02580 from the comparison between Williams 82 and PI 88788, one synonymous SNP in the first exon, one in the intron and one in the 3’ untranslated region (UTR) (Figure 1B). Glyma18g02590 possesses more SNP/InDels. The alignment of both published sequences (alleles in black) and our sequencing result of 11 soybean lines (alleles in red) revealed five SNPs in the 6th exon and two SNPs in the last exon. All of them lead to amino acid changes in the protein. Three SNPs from the 5’UTR of Glyma02610 have also been demonstrated between Williams 82 and PI 88788.

**Figure 2:**
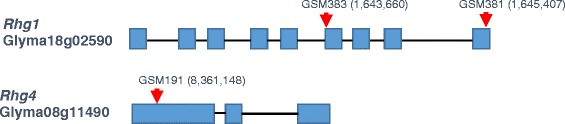
Positions of three SNPs in Glyma18g02590 and Glyma08g11490 at *Rgh1* and *Rhg4* loci, respectively. GSM numbers were used to designate SNP markers. Blue squares represent exons and black lines represent introns and UTRs. The genome coordinates (Database: Gmax_275_v2.0.softmasked) of each SNP are shown.

To evaluate which SNPs correlate with known SCN reactions of soybean lines, Kompetitive Allele Specific PCR (KASP) assays were developed and tested for all 13 SNPs at the *Rhg1* locus and two previously identified SNPs (Figure 1A) at *Rhg4* locus [14] using 27 soybean lines with known SCN reactions. Two SNPs from Glyma08g11490 are associated with the *Rhg4* resistance allele, and they are completely linked (data not shown). Three SNPs in Glyma18g02580 and two SNP/InDels in the last exon of Glyma18g02590 are associated with the *Rhg1* resistance allele, and all five markers are completely linked. Five SNPs in the 6th exon of Glyma18g02590 could distinguish the sources of Peking-type and PI88788-type resistance. However, three SNPs identified in the 5’-UTR of Glyma18g02610 showed no correlation with SCN resistance. Three SNPs were selected to provide genotype information for the selection of SCN resistance (Figure 2, Table 1), which were designated as GSM383, GSM381 and GSM191. GSM381 and GSM383 are located in the 6th and last exons of Glyma18g02590 at the *Rhg1* locus, respectively, while GSM191 is in the first exon of Glyma08g11490. The KASP assays of three SNPs performed with 27 soybean lines exhibited a good clustering of the homozygous alleles, suggesting that it is feasible to employ those markers for selection.

**Table 1:** **Haplotype alleles at** ***Rhg1*** **and** ***Rhg4*** **loci and source identification in 27 soybean lines** ^**‡**^

**Name**	**Markers at** ***Rhg1*** **locus**		**Marker at** ***Rhg4*** **locus**	**Reaction to SCN Race 3**	**Resistance type**
	**GSM381 (Gm18: 1645407)** ^**†**^	**GSM383 (Gm18: 1643660)**	**GSM191 (Gm08: 8361148)**		
PI548655 (Forrest)	G	G	G	R	Peking
PI548402 (Peking)	G	G	G	R	Peking
PI89772	G	G	G	R	Peking
PI90763	G	G	G	R	Peking
PI437654	G	G	G	R	Peking
PI548316	G	C	C	R	PI 88788
PI88788	G	C	C	R	PI 88788
PI209332	G	C	C	R	PI 88788
PI603372	G	C	C	R	PI 88788
PI603587A	G	C	C	R	PI 88788
PI603384	G	C	C	S	susceptible
PI97094	G	C	C	S	susceptible
PI548667	T	C	C	S	susceptible
PI518671 (Williams 82)	T	C	C	S	susceptible
PI567359	T	C	C	S	susceptible
PI567368	T	C	C	S	susceptible
PI567481	T	C	C	S	susceptible
PI602991	T	C	C	S	susceptible
PI603357	T	C	C	S	susceptible
PI603420	T	C	C	S	susceptible
PI603428C	T	C	C	S	susceptible
PI603502A	T	C	C	S	susceptible
PI603656	T	C	C	S	susceptible
PI567568B	T	C	C	S	susceptible
PI587993	T	C	C	S	susceptible
PI594770A	T	C	C	S	susceptible
PI594773	T	C	C	S	susceptible

^‡^Lines were used by Cook et al. [12] and Liu et al. [14].

^†^Numbers represent the genome coordinate based on the Database: Gmax_275_v2.0.softmasked.

### Haplotype comparison of resistance sources

**Figure 3:**
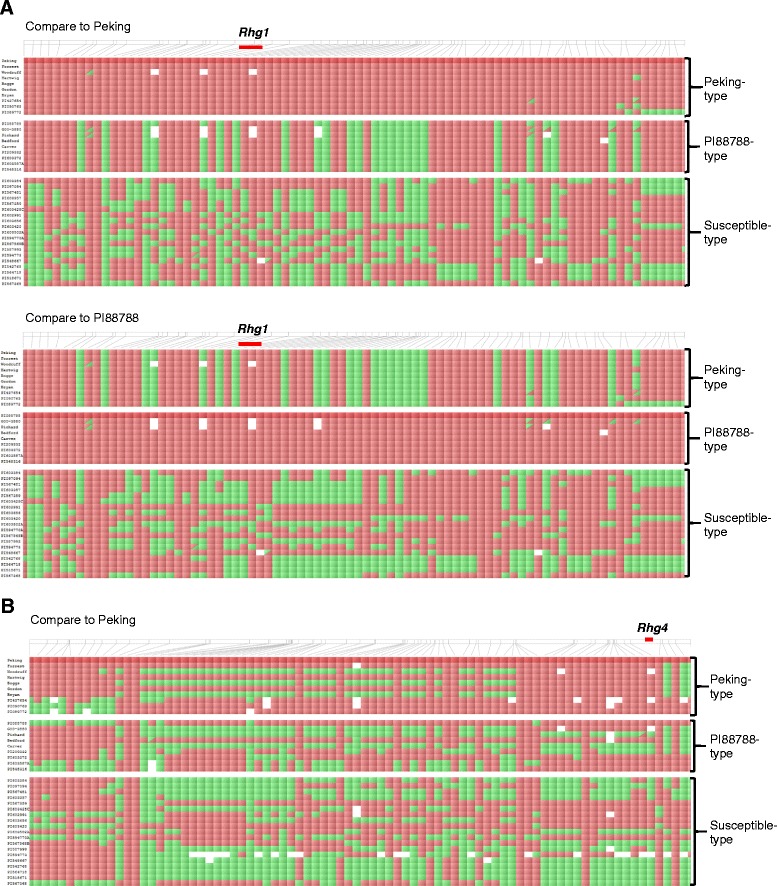
Haplotype analysis at *Rhg1* and *Rhg4* loci using Soy50k SNP Infinium chip data. Twenty-seven soybean plant introductions and nine cultivars, Woodruff, Hartwig, Boggs, Gordon, Bryan, G00-3880, Prichard, Bedford and Carver were used. **A**. Haplotype analysis at *Rhg1* locus. SNPs alleles at the *Rhg1* region on Chr18 were compared to Peking and PI 88788, respectively. A 788 kbp region (Coordinate of 1,266,210-2,054,952 on Chr18 based on Gmax_275_v2.0.softmasked) consisting of 82 SNPs is shown in the graph. **B**. Haplotype analysis at *Rhg4* locus. SNPs alleles at the *Rhg4* region on Chr08 were compared to Peking. A 1.5Mbp region (Coordinate of 6,903,359-8,399,514 on Chr08 based on Gmax_275_v2.0. softmasked) consisting of 82 SNPs is shown here. The reference line is shown in dark red. Red squares represent same alleles as the reference and green squares represent alleles that are different from the reference. The position of the locus and the resistance types are as indicated.

Haplotype analysis was performed using the Soy50k SNP Infinium chip data of 27 lines and nine cultivars including Woodruff, Hartwig, Boggs, Gordon, Bryan, G00-3880, Prichard, Bedford and Carver. The *Rhg1* region was compared between Peking and the Peking-type lines: Forrest, Woodruff, Hartwig, Boggs, Gordon, Bryan, PI 89772, PI 90763 and PI 437654, revealing that these lines have same haplotypes to Peking at the *Rhg1* locus as large as 5.9 Mbp. A 788 kb haplotype window block was shown in Figure 3A. Hartwig, PI 89772, PI 90763 and PI 437654 showed a relatively small haplotype window (710 kb) when compared to Peking. Woodruff had a larger haplotype block that is about 3.8Mbp and over 5.9Mbp was observed in Forrest, Gordon and Bryan. However, PI 88788 and other susceptible-type lines showed no introgression from Peking at the *Rhg1* locus. All eight PI 88788-type lines, G00-3880, Prichard, Bedford, Carver, PI 548316, PI 209332, PI 603372 and PI 603587A exhibited haplotype window blocks ranging from 1.6Mbp to 4.3Mbp, when compared to PI 88788 at the *Rhg1* region. Haplotype analysis of *Rhg4* locus on Chr08 indicated haplotype window blocks from Peking and nine Peking-type lines (Figure 3B), with a smaller region of 348 kb in Woodruff, Boggs and Bryan. However, the introgressed haplotype blocks are as large as 2.6Mbp in Forrest, Hartwig and Gordon. Examination of the pedigrees of these lines indicated that Peking is present somewhere in the extended pedigree of these six cultivars (http://www.ars-grin.gov/npgs/acc/acc_queries.html) [32-35]. These data are consistent with genotype indications of our three SNPs alleles and further substantiated the effectiveness of these SNP markers.

### Validation of SNP markers

**Figure 4:**
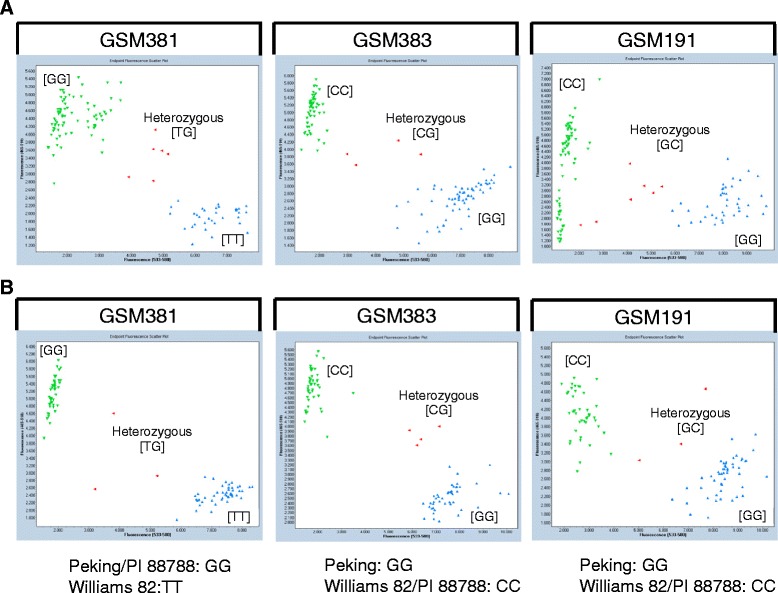
KASP graphs of three SNPs for validation and precision genotyping test population. **A**. KASP SNP graphs of 153 soybean lines from 2011–2013 USDA Southern Uniform Soybean Tests with known SCN race 3 reactions. **B**. KASP SNP graphs of F_5_-derived RILs from the population derived from G00-3213 x LG04-6000.

To validate the SNP markers with more diverse lines, we explored the data from the previous three years’ USDA Uniform Soybean Tests for Southern States where the phenotypes of SCN race 3 are available for all the tested lines. We requested a total of 153 lines from eight institutions and performed KASP assays on these lines with SNP markers, GSM381, GSM383 and GSM191 (Figure 4A). The SNP genotypes, SCN race 3 reactions and pedigrees were listed (Additional file 1: Table S1). The rating of SCN phenotype is on a scale of 1 to 5 based on the number of cysts, thus, lines with ratings smaller than 3 were considered as resistant and lines with ratings equal to or greater than 3 as susceptible. A strong accordance was observed between SNP genotypes and reactions to SCN race 3 for 145 germplasm lines with an exception of 10 lines (successful rate > 93%). Due to the large discrepancy of ratings for same line between two years, eight lines were excluded for analysis. Single factor analysis using General Linear Model (GLM procedure, SAS 9.3) showed a significant correlation between SCN resistance phenotypes and genotypes consisted of three SNP markers (R^2^ = 0.69, P < 0.001). Moreover, we have examined the extended pedigrees (data not shown) of these lines with resistance *Rhg1* allele (GSM381 = G) to identify the sources of resistance (Additional file 1: Table S1). The SNP genotypes are in complete agreement with the resistance sources based on extended pedigrees: Peking-source with a G allele at GSM383 and PI 88788-source with a C allele, indicating that GSM383 is a functional SNP to differentiate Peking and PI 88788 sources for the *Rhg1* resistance allele.

**Figure 5:**
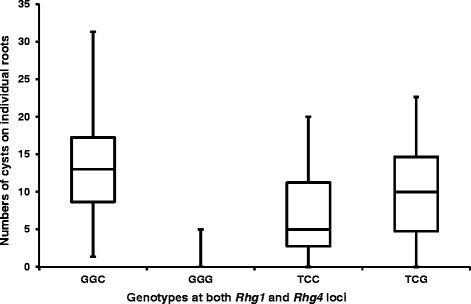
Reactions of race3 to different combinations of SNP marker alleles for 150 F_5_ families of G00-3213 x LG04-6000. *Rhg1* and *Rhg4* genotypes are represented as combinations of alleles of SNP markers, GSM381, GSM383 and GSM191. Boxes show median, 25% to 75% of the data and whiskers extend to minimum and maximum of the data.

We also performed a precision genotyping test on a bi-parental population of F_5_-derived Recombinant inbred lines (RILs) from G00-3213 x LG04-6000. The line G00-3213 carries a Peking-source *Rhg1* and *Rhg4* resistance alleles (GSM381 = G, GSM383 = G, and GSM191 = G) and is resistant to SCN race 3, while LG04-6000 is a susceptible line with T, C and C alleles for GSM381, GSM383, and GSM191, respectively. Three replicates of 150 RILs with two parents were arranged in a randomized complete block design in greenhouse and each entry was genotyped with three SNP markers (Figure 4B). Ten weeks after inoculation, phenotyping was carried out by counting the numbers of cysts on the roots of each individual plant. Resulting phenotypes demonstrated that combination of the resistance *Rhg1* (GSM381 = G and GSM383 = G) and resistance *Rhg4* alleles (GSM191 = G) brought a resistant phenotype with only 0.4 cyst/plant and all the other combinations exhibited a more susceptible phenotype (Figure 5, Table 2), suggesting that these three markers can be employed for the selection of SCN race 3 resistance from a population derived from Peking source. Single factor analysis using a GLM procedure in SAS 9.3 was performed for three subsets individually and combined. All of them showed a significant correlation between the average number of cysts and genotypes of three SNPs, GSM381, GSM383, and GSM191 (P < 0.0001), with an R-square of 0.64, 0.40, 0.24, and 0.3 for RIL sets 1, 2, 3 and combined, respectively. The different R-square values among sets might be due to the conditions at different inoculation times.

**Table 2:** **Genotypes of SNP markers and average number of cysts for 150 F**_**5**_ **families of G00-3213 x LG04-6000**

**SNP marker genotypes**			**Average numbers of cysts** ^**†**^
**GSM381**	**GSM383**	**GSM191**	
GG	GG	CC	11.1 b
GG	GG	GG	0.40 a
TT	CC	CC	7.0 b
TT	CC	GG	10.0 b

^†^Means followed by a different letter are significantly different at a probability level of 0.01.

## Discussion

The genetic basis of resistance to SCN is complex in soybean, not only because resistance to SCN is multi-genic, but also because different sets of genes/ Quantitative trait loci (QTLs) confer resistance to the diverse populations of SCN due to their genetic heterogeneity. Field populations of soybean cyst nematode have been classified into 16 races based on their ability to develop on resistant cultivars [6]. Resistance to SCN is race-cultivar specific [36], for example, soybean cultivars derived from Peking source are commonly claimed to be resistant to race 1 and 3, however, the cultivars with SCN resistance from PI 88788 are usually shown to be resistant to race 3 and 14 [37,38]. A growing body of evidence has shown different defense loci that underlie resistance to different races. The *Rhg1* locus on Chr18 is necessary for resistance to all SCN races [39]. Many other cultivar-race specific QTLs have been demonstrated in resistance sources, such as *Rhg4* for race 1 and 3, *Rhg3* and *Rhg5* for race 2 and *Rhg2* for race 5 and 14 [15,38,40,41]. Although two functional SNPs have been identified for *Rhg4* locus [14], no high-throughput marker assays have been reported to date. Moreover, several SNPs have been found at *Rhg1* locus [16], but no specific functional SNPs as well as detection assays have been reported to associate with the resistance allele, nor the SNPs to differentiate sources of resistance. Built on the previous discoveries of copy number variation, gene dosage, and DNA methylation at the *Rhg1* locus by Cook et al. [16] and SNPs at the *Rhg4* locus by Liu et al. [14], in this study, we have developed SNP marker assays, GSM381, GSM383, and GSM191 at *Rhg1* and *Rhg4* locus for the selection of resistance alleles, (Figure 2). The SNPs reside in the coding sequences of resistance genes (Glyma18g02590 and Glyma08g11490) and also introduce changes in protein sequences, thus they can be considered as functional markers for SCN race 3 resistance and applied in high throughput marker-assisted selection with high accuracy. Similarly, the knowledge of additional loci along with the genome sequence of soybean may be used in the marker development for resistance to different SCN races.

PI 88788 has been the primary choice in soybean cultivar development as a source for SCN resistance [1], however, SCN populations have demonstrated the capability to mutate and recombine to new races, which can overcome the soybean resistance derived from PI 88788 [36]. Reports have shown that PI 88788 has not been providing sufficient protection against SCN in some fields [42-45]. Thus, additional resistance sources are desired to be used for developing new resistant cultivars and Peking is believed to be an attractive option. Although SNP GSM381 discriminates resistant and susceptible *Rhg1* alleles, it does not distinguish PI 88788- and Peking-type *Rhg1* loci. Thus, to identify the sources of resistance, we demonstrated that one SNP marker at *Rhg1* locus, GSM383 was able to distinguish Peking from PI 88788 loci for most soybean germplasm (Figure 4 and Additional file 1: Table S1). This SNP can be employed in differentiating Peking from PI 88788 sources for the *Rhg1* allele for breeding programs.

The successful prediction rate for three SNP markers at *Rhg1* and *Rhg4* loci is over 93% in this study (Figure 4 and Additional file 1: Table S1). A few lines (10 lines from USDA Uniform Test (Additional file 1: Table S1) and two lines from 27 validation lines (Table 1)) did not show a correlation between the expected phenotypes and the resistance SNP alleles. A number of factors may result in the discrepancy between genotyping and phenotyping results, including 1) phenotyping/ or seed source errors: some of the lines have been tested for only one year, and one-time phenotyping of SCN resistance may not be definitive; 2) sources other than PI 88788 and Peking may be providing resistance; and 3) the complexity of the *Rhg1* region is not fully understood. A future study will be designed to understand why the lines with the resistance genotypes did not have a resistance reaction to SCN race 3. The 31.2 kb region at *Rgh1* locus is repeated in resistant genotypes [12], and the sequences of the individual copies are also variant within a single genotype, which makes marker development challenging for the *Rhg1* locus. We also tested marker performance with artificial heterozygous DNA, where PI 88788/ Peking and Williams 82 DNA were mixed at 1:1 ratio. Although the artificial heterozygous allele cluster was located a little closer towards the resistant homozygous allele cluster in the KASP SNP graphs (data not shown) because of the gene repeats in resistant lines, the alleles can still be called clearly. Therefore, the functional SNP markers that we identified can be used not only to select the resistance allele, but also to differentiate resistance sources, which were also confirmed by analysis of the *Rhg1* region with Soy50k SNP Infinium chip data using 38 soybean lines (Figure 3A).

A recent study of the *Rhg1* locus revealed that copy number variation of the 31 kb DNA fragment, DNA sequence variation, and differentially methylated patterns were correlated with the *Rhg1*-mediated resistance to SCN [12,16]. These discoveries may also offer additional selection strategies for the *Rhg1* allele, in addition to the functional SNP detection assays we developed. Cook et al. [16] explored the whole genome data for *Rhg1* locus in 42 soybean lines, and multiple lines are in common in both studies. In this study, we got the same C-terminal protein sequence of Glyma18g02590 for both low-copy and high-copy *Rhg1* as described in the previous study ([16] Figure 3B), indicating that GSM 381 maybe also exist in their DNA sequences. Interestingly, they identified a novel splice isoform of Glyma18g02590 cDNA in Peking-type *Rhg1* lines, however, we did not detect this isoform in our cDNA sequencing, probably due to the low copy number of this isoform and use of a different sequencing method. Thus, more studies are needed to investigate the *Rhg1* locus in various soybean lines for new insights into the development of improved selection approaches.

Soybean cyst nematode is estimated to cause more than one billion dollars yield loss annually in the USA [2], and thus nematode resistance is of tremendous interest to soybean breeders and growers. The SNP markers we identified could be used to guide the screening of soybean germplasm for SCN resistance for both Peking and PI 88788 sources, as well as to improve the efficiency and precision of selection for SCN resistance in breeding programs. If the known resistance source was used as one of the parents in a cross, only GSM381 is needed to select for PI 88788-type resistance, while both GSM383 and GSM191 are required to select for Peking-type resistance. Moreover, the KASP assays that we developed were PCR-based assays and robust in detecting the resistance allele and separating heterozygotes from homozygotes and thus can be used in high-throughput selection of SCN resistance. Finally, with aid of molecular markers, multiple SCN resistance genes can be pyramided into elite soybean genotypes in a more timely and labor-efficient manner.

## Conclusions

The genomic sequence of Glyma08g11490 at *Rhg4* locus in 28 soybean lines and Glyma18g02590 at *Rhg1* locus in 11 lines, along with the known reactions to soybean cyst nematode race 3 of these lines, allowed us to identify functional SNPs and develop robust KASP SNP assays for marker-assisted breeding. Using a panel of 153 soybean germplasm and a segregating bi-parent population, we have validated the usefulness of these three SNP markers, two at *Rhg1* locus and one at *Rhg4.* The results of this work demonstrated that these markers could be applied for high throughput selection of SCN resistance from PI 88788 and Peking sources and for accelerating breeding of SCN resistance in an accurate and efficient manner.

## Methods

### Plant materials

Twenty-seven soybean lines with known SCN phenotypes that were used in both studies by Cook et al. [12] and Liu et al.[14] (Listed in Table 1) were selected for the initial sequencing and SNP identification. The seeds were obtained from the USDA Soybean Germplasm Collection (Urbana, IL) and grown in the greenhouse. Leaf tissue was collected from 15 plants of each line for bulk DNA extraction.

One hundred and fifty-three soybean lines that were entered into the USDA Uniform Soybean Tests for Southern States from 2011 to 2013 with known SCN reactions were selected for SNP marker validation. These lines were kindly provided by their respective breeders. The seed was planted in 32 oz cups in the greenhouse with 15 seed/line. Twelve leaves were pooled from 12 plants with one from each plant and samples were freeze-dried for 48 hours to be used for DNA extraction.

The 150 F_5:6_ RIL lines were derived from the cross of G00-3213 x LG04-6000 using a single seed descent method. G00-3213 is a high-yielding line with nematode resistance developed at the University of Georgia. LG04-6000 (Reg. No. GP-379, PI 664025) is known to be genetically diverse and high-yielding line that was developed and released by the USDA-Agricultural Research Service and the Illinois Agricultural Experiment Station, Urbana IL.

### Sequencing and SNP identification

To discover SNPs at the *Rhg1* locus, the genomic sequences of three genes, Gm18g02580, Gm18g02590 and Gm18g02610 from Williams 82 were obtained from Phytozome (http://www.phytozome.net/). The 31.2 kb region from PI 88788 and the cDNA sequence of Gm18g02590 were acquired from NCBI (JX907806) and the publication of Matsye et al. [31], respectively. DNA alignment was carried out using Geneious version 5.5.7 and potential SNPs were identified. To obtain additional SNPs at the *Rhg1* locus, genomic DNA and cDNA of Gm18g02590 was sequenced from 11 soybean lines (Forrest, PI 437654, Peking, PI 89772, PI 90763, PI 88788, PI 548316, PI 209332, Williams 82, PI 603428C and Essex) and four lines (Forrest, Essex, Williams 82 and PI 88788) with known phenotype reactions, respectively. Four pairs of specific primers (Additional file 2: Table S2) were designed according to Williams 82 genomic sequence of Gm18g02590. At least two independent PCR amplifications were performed for each primer pair. Individual PCR products were cloned into the pGEM-T vector and 10 clones were randomly selected for each PCR amplification and sequenced. The entire gene sequences were obtained from the alignment of the corresponding four fragments and blasted with Williams 82 genomic sequence.

To identify the SNPs at the *Rhg4* locus, the genomic DNA of Gm08g11490 was sequenced from 28 lines (above 27 lines and RESSEQ). The collection of soybean lines used in sequencing was obtained from the USDA Soybean Germplasm Collection (Urbana, IL). The soybean seed was planted in the greenhouse of the Horticultural Research Center at Southern Illinois University, Carbondale, IL. Young leaf tissue of the soybean lines was harvested to extract DNA using the DNeasy Plant Mini Kit (Qiagen). According to the genome reference sequence of Williams 82, the specific primers were designed to amplify the genomic DNA fragments of *GmSHMT* and *GmSNAP* with a 38 cycles of PCR amplification at 94°C for 30 s, 50-60°C for 30 s and 72°C for 90 s. The PCR products were purified using the QIAquick PCR Purification Kit (Qiagen). Then, the purified PCR fragments were sequenced at GENEWIZ Company (www.genewiz.com), and the sequences were aligned. Primers used in sequencing were listed in Additional file 2: Table S2.

### Haplotype analysis

To understand the genetic variation at both the *Rhg1* and *Rhg4* loci, Soy50K SNP Infinium chip data for 27 above soybean lines and nine cultivars, Woodruff, Hartwig, Boggs, Gordon, Bryan, G00-3880, Prichard, Bedford and Carver were obtained from Soybase (www.soybase.org) or our laboratory database at the University of Georgia, respectively. The SNPs on Chr18 and Chr08 for these 36 lines were imported into Flapjack software [46] along with the corresponding marker genetic map file for visualization of the haplotypes around *Rhg1* and *Rhg4* loci. The color scheme of “By similarity to line” was applied to compare to SNP alleles of Peking and PI 88788 and the region of introgression from sources was determined by the haplotype markers with different alleles to the reference lines.

### KASP assays design and SNP validation

To test the association of SNPs with the reactions to SCN race 3 and develop robust markers for high throughput selection, Kompetitive Allele Specific PCR (KASP) assays were developed and tested for all the identified SNPs using above 27 soybean lines with known SCN phenotypes. The assays were performed as previously described [47]. Three SNPs, GSM381 (in Glyma18g02590, G/T), GSM383 (in Glyma18g02590, G/C) and GSM191 (in Glyma08g11490, G/C), were then selected for marker validation. Primer sequences are listed in Table 3. KASP assays were run with 4 μL reaction system including 2 μL low rox KASP master mix (KBiosciences, Herts England), 0.106 μL of primer mix (0.318 μL of each primer at final concentration) and 2 μL of 10-25 ng/μl genomic DNA. The PCR conditions for KASP marker assay was 94°C for 15 min, followed by 10 cycles of touch down PCR from 68°C to 60°C with 0.8°C decrease per cycle, then followed by 30 cycles of 94°C for 20 s and 57°C for 1 min. . PCR fluorescent endpoint readings were performed using the Light Cycler® 480 Real-Time PCR System (Roche, Germany).

**Table 3:** **KASP assay primer sequences of three SNPs, GSM381, GSM383 and GSM191**

**Locus**	**Gene**	**Marker**	
*Rhg1*	Gm18g02590	GSM381	**FAM_primer**: GAAGGTGACCAAGTTCATGCTAGCCAAAGAACTTGAGSAGBATGAG
			**VIC_Primer**: GAAGGTCGGAGTCAACGGATTAGCCAAAGAACTTGAGSAGBATGAT
			**Common reverse primer**: CAAACAATAGGTCCAACCACCA
*Rhg1*	Gm18g02590	GSM383	**FAM_primer**: GAAGGTGACCAAGTTCATGCTATCTGCMAACTCTGTAAAGAGGAC
			**VIC_Primer**: GAAGGTCGGAGTCAACGGATTATCTGCMAACTCTGTAAAGAGGAG
			**Common reverse primer**: GCTGTCCAGTCTCCAGAAGTGAA
*Rhg4*	Gm08g11490	GSM191	**FAM_primer**: GAAGGTGACCAAGTTCATGCTCATCATGGGGCTAGATCTCCC
			**VIC_Primer**: GAAGGTCGGAGTCAACGGATTCATCATGGGGCTAGATCTCCG
			**Common reverse primer**: TAGCCGGTGGTGGAGTTTACC

The seeds of 153 soybean lines were planted in 32 oz cups in the greenhouse with 15 seeds per line. One leaf from each of 12 plants were pooled and leaf samples were freeze-dried for 48 hours. DNA was isolated using a 96-well plates as described in [48]. KASP assays were performed for three SNP markers with Peking (resistant) and Williams 82 (susceptible) as controls. Associations of the markers with SCN resistance were tested using single-factor analysis of variance with a General Linear Model (GLM) procedure in SAS 9.3 (SAS Institute, 2013) based on the known phenotype reactions to SCN race 3 from USDA Uniform Soybean Tests for Southern States.

The 150 F_5_-derived recombinant inbred lines (RILs) of G00-3213 x LG04-6000 were randomized and grown in the greenhouse along with the parents and controls for phenotyping and tissue sampling for DNA extraction. DNA extraction procedures and KASP assays for three SNP markers were performed as described above. Associations of the markers with SCN resistance were tested using single-factor analysis of variance with a GLM procedure in SAS 9.3 (SAS Institute, 2013) based on means of the cyst numbers from three replicates for each subset separately and combined.

### SCN phenotyping assays

The phenotypic data for reactions to SCN race 3 and direct pedigrees of soybean lines were acquired from 2011–2013 USDA Uniform Soybean Tests for Southern States [49-51]. The extended pedigrees were acquired by searching the pedigree of immediate parents on GRIN and/or past USDA Uniform Soybean Tests for Southern States. The screenings for SCN reactions were conducted in the USDA-ASR-SEA greenhouse, Jackson, TN. One seed of each line was planted in sterile soil mix in a three inch sterile clay pot. Screening for SCN was done with HG type 0 (race 3) in 2011, HG type 5.7 (race 3) in 2012 and HG type 5.7 (race 3) in 2013. Three replications were used for each entry per HG Type. At the time of planting, approximately 2,000 eggs of the nematode population (HG Type) extracted from crushed SCN females were added to each pot. Thirty days (+/−1 day) after planting, plants were rated based on the number of cysts on the roots. The rating scale was as follows: 1 = 0-5 cysts; 2 = 6-10 cysts; 3 = 11-20 cysts; 4 = 21-40 cysts; and 5= > 40 cysts [49]. Homogeneous nematode populations that had been cultured in the greenhouse for several reproductive cycles were used. The HG Type was confirmed using established indicator lines [7].

The 150 F_5_-derived RILs of G00-3213 x LG04-6000 were divided into three subsets for greenhouse phenotyping, each with 50 lines and parents. Parental lines G003213 and LG04-6000, and resistant and susceptible controls, Bryan [34] and Haskell [52], were also included twice in each set. Three replications for each line were arranged in a randomized complete block design and three seeds for each replication were planted into a 2.5 cm-diameter cone-tainer. One week after planting, the plants were thinned to one and the roots were inoculated with 4,000 SCN race 3 eggs using a digital dispensing pump. Ten weeks after inoculation, the numbers of cysts were counted on each individual plant.

## Additional files

Additional file 1: Table S1. Marker alleles, pedigrees and phenotypes for SCN race 3 in 153 soybean lines entered into the USDA Uniform Soybean Tests for Southern States. Additional file 2: Table S2. Primers used for sequencing of GmSHMT and GmSNAP.

## Abbreviations

SCN: Soybean cyst nematode
SSR: Simple sequence repeat
*Rhg*: Resistance to *H. glycines*
Chr: Chromosome
SNP: Single nucleotide polymorphism
InDel: Insertion/ deletion
KASP: Kompetitive Allele Specific
PCR: Polymerase chain reaction
HG: *Heterodera glycines*
RIL: Recombinant inbred lines
GLM: General linear model
RFLP: Restriction fragment length polymorphism
SHMT: Serine hydroxymethyltransferase
UTR: Untranslated region
QTL: Quantitative trait loci
