# Supplementary material for: SNP identification and marker assay development for high-throughput selection of soybean cyst nematode resistance
Source: BMC Genomics. 2015 Apr 18;16(1):314. doi: 10.1186/s12864-015-1531-3 (PMC4407462; doi:10.1186/s12864-015-1531-3)
Supplement: Additional file 1: Table S1. — Marker alleles, pedigrees and phenotypes for SCN race 3 in 153 soybean lines entered into the USDA Uniform Soybean Tests for Southern States. [file 12864_2015_1531_MOESM1_ESM.docx]

**Adittional file 1: Table S1.** Marker alleles, pedigrees and phenotypes for SCN race 3 in 153 soybean lines entered into the USDA Uniform Soybean Tests for Southern States

| **Line name** | **Institution** | **Pedigree** | **Reaction SCN Race 3**† | SNP Marker allele‡ | | | **Year in the test** | **Source of Resistance based on extended pedigree** |
| --- | --- | --- | --- | --- | --- | --- | --- | --- |
|  |  |  |  | **GSM381** | **GSM383** | **GSM191** |  |  |
| G05-1102 RR | University of Georgia | G98-1420 x H7242 RR | 1.0/1.0 | G | G | G | 2011/2012 | Peking |
| G06-2460 RR | University of Georgia | G98-1420 x H7242 RR | 1.0/1.0 | G | G | G | 2011/2012 | Peking |
| R07-6669 | University of Arkansas | Lonoke x R00-33 | 1.0/3.6 | G | C | C | 2012/2013 | Peking + PI 88788 |
| R04-522 | University of Arkansas | Lonoke x P9594 | 1.0 | G | C | C | 2011 | Peking + PI 88788 |
| G04-2215RR | University of Georgia | G96-2272 x H7242 RR | 1.0/5.0 | G | G | G | 2011/2012 | Peking |
| G08-3795RR | University of Georgia | G99-2678 x P97M50 | 1.0 | G | C | G | 2012 | PI 88788 |
| G07-2879 RR | University of Georgia | G00-3083 x AGS758RR | 1.0/1.0 | G | G | G | 2011/2012 | Peking |
| G08-5122 RR | University of Georgia | N97-9658 x G02-G176376 | 1.0/1.6 | G | G | G | 2012/2013 | Peking |
| G06-3182 RR | University of Georgia | G99-4158 x P97M50 | 1.0/1.0/1.0 | G | G | G | 2011/2012/2013 | Peking |
| G03-1187 RR | University of Georgia | G95-346 x H7242 RR | 1.0 | G | G | G | 2011 | Peking |
| TN09-029 | University of Tennessee | Fowler × Anand | 1.0 | G | G | G | 2012 | Peking |
| SC07-786RR | Clemson University | SC01-786RR x G00-3213 | 1.0/1.0/1.6 | G | G | G | 2011/2012/2013 | Peking |
| SC07-1518RR | Clemson University | SC01-809RR x G99-3211 | 1.0/1.0/3.1 | G | G | G | 2011/2012/2013 | Peking |
| SC07-1490RR | Clemson University | SC01-809RR x G99-3211 | 1.0/1.0/1.2 | G | G | G | 2011/2012/2013 | Peking |
| SC07-108RR | Clemson University | N97-9658 x SC01-783RR | 1.0/3.0/4.7 | G | C | G | 2011/2012/2013 | Peking + PI 88788 |
| SC07-1029RR | Clemson University | G00-3213 x SC00-643RR | 1.0/1.0 | G | G | G | 2011/2012 | Peking |
| SC06-708RR | Clemson University | SC01-809RR x G99-3211 | 1.0/1.0 | G | G | G | 2011/2012 | Peking |
| SC06-676RR | Clemson University | SC01-809RR x G99-3211 | 1.0/1.0 | G | G | G | 2011/2012 | Peking |
| SC06-301RR | Clemson University | N97-9658 x SC01-783RR | 1.0/5.0 | G | C | C | 2011/2012 | Peking + PI 88788 |
| SC05-642RR | Clemson University | SC00-603RR x SC94-1573 | 1.0 | G | G | G | 2011 | Peking |
| SC01-803 RR | Clemson University | SC92-2482 x {SC92-2482 x [HAGOOD x (HAGOOD x BC1RES NIKRR)]} | 1.0/1.0 | G | G | G | 2011/2012 | Peking |
| R09-1970 | University of Arkansas | R00-1076 x S99-2281 | 1.0 | G | G | G | 2013 | Peking |
| R07-6654 | University of Arkansas | Lonoke x R00-33 | 1.0 | G | C | C | 2012 | Peking + PI 88788 |
| R07-10244 | University of Arkansas | S99-2281 x UA 4805 | 1.0 | G | G | G | 2011 | Peking |
| R07-10231 | University of Arkansas | S99-2281 x UA 4805 | 1.0 | G | G | G | 2011 | Peking |
| JTN-5503 | USDA-ARS, Jackson, TN | Fowler x Manokin | 1.0 | G | G | G | 2011 | Peking |
| JTN-5208 | USDA-ARS, Jackson, TN | S96-2641 x S97-1688-8-LOAM02 | 1.0 | G | G | G | 2011 | Peking |
| JTN-5108 | USDA-ARS, Jackson, TN | S95-1908 x Bolivar | 1.0/1.0 | G | G | G | 2011/2012 | Unknown |
| JTN-4607 | USDA-ARS, Jackson, TN | LS94-3207 x S95-1908 | 1.0/1.0 | G | G | G | 2011/2012 | Peking |
| JTN-4408 | USDA-ARS, Jackson, TN | S97-1753 x V94-0198 | 1.0 | G | G | G | 2011 | Peking |
| JTN-4307 | USDA-ARS, Jackson, TN | S97-1688 x V94-0198 | 1.0/1.0 | G | G | G | 2012/2013 | Peking |
| V05-2664 | Virginia Tech | R95-2210 x V96-0332 | 1.0/3.0 | G | C | C | 2011/2012 | Peking + PI 88788 |
| S10-10368 | University of Missouri | S04-8882 x S05-11400 | 1.0 | G | G | G | 2013 | Peking |
| G07-1185 RR | University of Georgia | G00-3213 x(Boggs RR(2) x N97-9658) | 1.0/1.0 | G | G | G | 2011/2012 | Peking |
| G08-3282 RR | University of Georgia | G00-3209 x G03-952 RR | 1.0 | G | G | G | 2012 | Peking |
| G08-2869 RR | University of Georgia | G00-3083 x AGS758RR | 1.0 | G | G | G | 2012 | Peking |
| G07-3557 RR | University of Georgia | G00-3213 x P97M50 | 1.0/1.0 | G | G | G | 2011/2012 | Peking |
| JTN-5110 | USDA-ARS, Jackson, TN | J98-32 x Anand | 1.0/1.0/1.0 | G | G | G | 2011/2012/2013 | Peking |
| G08-4200 RR | University of Georgia | N97-9658 x BOGGS RR | 1.0/1.0 | G | G | G | 2012/2013 | Peking |
| G10PR-224R2 | University of Georgia | G00-3213 x NC7308A1-COYN | 1.0 | G | G | G | 2013 | Peking |
| N7003CN | USDA-ARS, Raleigh, NC | Cook × Anand | 1.0/4.0/1.5 | G | G | C | 2011/2012/2013 | Peking |
| R06-4475 | University of Arkansas | R98-3267F x R97-1832 | 2.0/5.0 | G | C | C | 2012/2013 | Peking + PI 88788 |
| G05-4237RR | University of Georgia | Prichard-RR x G94-3117 | 2.0/2.0 | G | C | G | 2011/2012 | PI 88788 |
| TN08-114 | University of Tennessee | TN02-303 × S98-1375 | 3/2.7 | G | G | C | 2012/2013 | Peking |
| TN08-109 | University of Tennessee | TN02-303 × S98-1375 | 2.0/2.0 | G | G | C | 2011/2012 | Peking |
| SC07-150RR | Clemson University | N97-9658 x SC00-643RR | 2.0 | G | G | C | 2011 | Peking |
| SC06-291RR | Clemson University | SC98-1930 x SC00-892RR | 2.0/4.3 | G | C | G | 2012/2013 | PI 88788 |
| SC06-007RR | Clemson University | SC98-1850 x SC00-892RR | 2.0/4.3 | G | G | G | 2012/2013 | Peking |
| R09-400 | University of Arkansas | R00-1076 x BA 743303 | 2.0 | G | C | C | 2012 | PI 88788 |
| R08-47 | University of Arkansas | 5601T x R00-1940 | 2.0 | G | C | G | 2012 | Peking + PI 88788 |
| R07-5351 | University of Arkansas | LS96-1631 x R96-3427 | 2.0 | G | G | G | 2012 | Unknown |
| R07-1857 | University of Arkansas | R01-2373 x R01-315 | 2.0 | G | C | G | 2012 | PI 88788 |
| S05-11482 | University of Missouri | S99-2281 x S00-9985-03 | 2.0 | G | G | C | 2011 | Peking |
| S08-9727 | University of Missouri | S04-8952 x S03-1904 | 2.0 | G | C | C | 2012 | Unknown |
| S09-14199 | University of Missouri | S04-11681 x S04-12412 | 2.0 | G | G | G | 2012 | Unknown |
| JTN-5203 | USDA-ARS, Jackson, TN | R93-171 x Anand | 2.0/2.0/1.0 | G | G | C | 2011/2012/2013 | Peking |
| G08-3279 RR | University of Georgia | G00-3209 x G03-952 RR | 2.0/4.0 | G | G | G | 2012/2013 | Peking |
| SC09-142RR | Clemson University | G00-3213 x SC00-643RR | 2.1 | G | G | G | 2013 | Peking |
| SC09-142RR | Clemson University | G00-3213 x SC00-643RR | 2.1 | G | G | G | 2013 | Peking |
| TN09- 47,169 | University of Tennessee | TN02-226 x MON RR2Y | 2.6 | G | G | C | 2013 | Peking |
| SC09-102RR | Clemson University | G00-3213 x SC00-643RR | 2.7 | G | G | G | 2013 | Peking |
| SC09-102RR | Clemson University | G00-3213 x SC00-643RR | 2.7 | G | G | G | 2013 | Peking |
| SC09-210RR | Clemson University | SC01-809RR x G99-3211 | 2.7 | G | G | G | 2013 | Peking |
| AGS606RR | AgSouth Genetics | Unknown, Commercial check | ../3.0/3.9 | G | C | C | 2011/2012/2013 | PI 88788 |
| TN09-004 | University of Tennessee | Fowler × Anand | 3.0/4.0 | G | G | C | 2011/2012 | Peking |
| TN08-113 | University of Tennessee | TN02-303 × S98-1375 | 3.0 | G | G | C | 2012 | Peking |
| SC09-092RR | Clemson University | N97-9658 x SC00-643RR | 3.0 | G | G | G | 2013 | Peking |
| R08-141 | University of Arkansas | R00-1076 x R00-1940 | 3.0 | G | C | G | 2012 | PI 88788 |
| R07-1826 | University of Arkansas | R00-1551 x R01-315 | 3.0/2.6 | G | G | G | 2012/2013 | Unknown |
| R05-3239 | University of Arkansas | Ozark x Anand | 3.0 | G | G | C | 2011 | Peking |
| S08-14117 | University of Missouri | R00-1194F x S04-5969RR | 3.0 | G | C | C | 2012 | PI 88788 |
| N05-7462 | USDA-ARS, Raleigh, NC | 5601T x N96-6809 | 3.0 | G | H | H | 2011 | Unknown |
| SC09-092RR | Clemson University | N97-9658 x SC00-643RR | 3.0 | G | G | G | 2013 | Peking |
| S08-9942RR | University of Missouri | R00-1194F x S03-383RR | 3.3 | G | C | C | 2013 | PI 88788 |
| R09-5088 | University of Arkansas | 5002T x UA 4805 | 3.4 | G | G | G | 2013 | Peking |
| TN09-44,420 | University of Tennessee | TN02-226 x MON RR2Y | 3.4 | G | G | C | 2013 | Peking |
| V08-1924 | Virginia Tech | S00-9925-10 x S00-9985-03 | 3.6 | G | G | C | 2013 | Peking |
| S10-2635RR | University of Missouri | S06-7084 x RR2-6851 | 3.8 | G | G | C | 2013 | Unknown |
| TN09-008 | University of Tennessee | Fowler × Anand | 4.0/1.1 | G | G | C | 2012/2013 | Peking |
| TN05-5018 | University of Tennessee | 5601T × 5002T | 4.0 | G | G | C | 2011 | Peking |
| R09-430 | University of Arkansas | BA 743303 x R00-684 | 3.0/3.1 | G | C | C | 2012/2013 | PI 88788 |
| R07-1810 | University of Arkansas | R00-1551 x R01-2346 | 4.0 | G | G | C | 2011 | Unknown |
| V05-2592 | Virginia Tech | R95-2210 x V94-0436 | 4.0 | G | G | C | 2011 | Peking |
| G10PR-86R2 | University of Georgia | G00-3880 x NC7308A1-COYN | 4.2 | G | C | C | 2013 | PI 88788 |
| G09-3202R2 | University of Georgia | G00-3880 x NC7308A1-COYN | 4.3 | G | C | H | 2013 | PI 88788 |
| SC09-052RR | Clemson University | N97-9658 x SC00-643RR | 4.3 | G | G | C | 2013 | Peking |
| SC09-039RR | Clemson University | N97-9658 x SC00-643RR | 4.3 | G | G | H | 2013 | Peking |
| SC09-052RR | Clemson University | N97-9658 x SC00-643RR | 4.3 | G | G | C | 2013 | Peking |
| G10PR-56444R2 | University of Georgia | G93-2225 x (G00-3213 x RR2Y) | 4.6 | G | G | C | 2013 | Peking |
| G10PR-56330R2 | University of Georgia | G93-2225 x (G00-3213 x RR2Y) | 4.8 | G | G | C | 2013 | Peking |
| V08-0136 | Virginia Tech | 99VPI-67 x S00-9925-10 | 4.9 | G | G | C | 2013 | Peking |
| SC06-051RR | Clemson University | SC98-1850 x SC00-892RR | 5.0 | G | G | C | 2012 | Peking |
| V05-2607 | Virginia Tech | R95-2210 x V94-0436 | 5.0 | G | G | C | 2011 | Peking + PI 88788 |
| AGS828RR | University of Georgia | PRICHARD-RR x SC96-1476 | 5.0 | G | C | G | 2013 | PI 88788 |
| G10PR-56264R2 | University of Georgia | G00-3213(2) x RR2Y | 5.0 | G | G | C | 2013 | Peking |
| S07-2680 | University of Missouri | S99-2281 x S02-6143 | 1.0/4.0 | T | C | G | 2011/2012 | Unknown |
| TCHM06-M-204 | North Carolina State University | Late maturing mutant in Holladay M3 | 2.0/5.0 | T | C | H | 2011/2012 | Unknown |
| TN11-4513 | University of Tennessee | TN02-226 x MON RR2Y | 3.0 | T | C | H | 2013 | Peking |
| R09-1589 | University of Arkansas | 5002T x R01-4752 | 3.0 | T | C | C | 2013 | Unknown |
| N7002 | USDA-ARS, Raleigh, NC | N7001 × Cook | 3.0/5.0 | T | C | C | 2011/2012 | Unknown |
| R03-1250 | University of Arkansas | PIO 9592 x KS4895 | 3.0 | H | C | C | 2011 | Unknown |
| V06-0855 | Virginia Tech | S96-2641 x V97-1549 | 3.0 | T | C | C | 2011 | Unknown |
| R09-4571 | University of Arkansas | DP4748 x S01-9794 | 3.3 | T | C | C | 2013 | Unknown |
| S09-9943 | University of Missouri | S04-10364 x LG04-5196 | 3.6 | T | C | C | 2013 | Unknown |
| R08-527 | University of Arkansas | UA 4805 x 5002T | 3.8 | T | C | C | 2013 | Unknown |
| NCC04-619 | North Carolina State University | N97-61 x TN96-64 | 4.0 | T | C | C | 2011 | Unknown |
| R08-1178 | University of Arkansas | S98-1375 x R97-1634 | 4.0 | H | H | C | 2012 | Unknown |
| R06-4433 | University of Arkansas | Lonoke x P9594 | 4.0 | T | C | C | 2011 | Unknown |
| R05-3817 | University of Arkansas | R96-3427 x 605 | 4.0 | T | C | C | 2011 | Unknown |
| R05-374 | University of Arkansas | Lonoke x DP4748 | 4.0/4.0 | T | C | H | 2011/2012 | Unknown |
| V05-2037 | Virginia Tech | 5002T x V99-3337 | 4.0 | T | C | C | 2011 | Unknown |
| V05-2326 | Virginia Tech | V95-0391 x N96-7211 | 4.0 | T | C | C | 2011 | Unknown |
| V06-0283 | Virginia Tech | V96-0340 x V94-1401 | 4.0 | T | C | C | 2011 | Unknown |
| S08-14087 | University of Missouri | R00-1194F x S04-5969RR | 4.0 | T | C | C | 2011 | Unknown |
| S08-17361 | University of Missouri | LG04-5196 x S00-9925-10 BS | 4.0 | T | C | C | 2011 | Unknown |
| OSAGE | University of Arkansas | Hartz 5545 x KS4895 | 4.0/4.0/.. | T | C | G | 2011/2012/2013 | Unknown |
| Dillon | North Carolina State University | Centennial x Young | 1.0/4.0/4.7 | T | C | G | 2011/2012/2013 | Unknown |
| NCC06-1090 | North Carolina State University | N99-8137 x TN99-117 | 3.0/4.0/4.1 | T | C | C | 2011/2012/2013 | Unknown |
| NC-ROY | USDA-ARS, Raleigh, NC | Holladay x Brim | 4.0/4.0/4.3 | T | C | C | 2011/2012/2013 | Unknown |
| N05-7432 | USDA-ARS, Raleigh, NC | N7002 x N98-7265 | 3.0/3.0/4.4 | T | C | C | 2011/2012/2013 | Unknown |
| N10-7121 | USDA-ARS, Raleigh, NC | ROY x 398833-BB | 4.2 | T | C | C | 2013 | Unknown |
| N10-7143 | USDA-ARS, Raleigh, NC | ROY x 398976-BB | 4.2 | T | C | C | 2013 | Unknown |
| TN08-100 | University of Tennessee | 5601T x PI417088 | 4.2 | T | C | C | 2013 | Unknown |
| N09-13671 | USDA-ARS, Raleigh, NC | N98-7961 x N02-8718 | 4.0/4.9 | T | C | C | 2012/2013 | Unknown |
| N8001 | USDA-ARS, Raleigh, NC | N7001 × Cook | 3.0/4.0/3.8 | T | C | C | 2011/2012/2013 | Unknown |
| S09-6201 | University of Missouri | S04-20912RR x S05-4630RR | 4.4 | T | C | H | 2013 | Unknown |
| R10-5721 | University of Arkansas | 5601T x R01-2195 | 4.5 | T | C | C | 2013 | Unknown |
| N09-13690 | USDA-ARS, Raleigh, NC | NC-ROY x N02-8718 | 4.5 | T | C | C | 2013 | Unknown |
| S09-13635 | University of Missouri | LG04-6863 x S04-10364 | 4.6 | T | C | C | 2013 | Unknown |
| TN11-5062 | University of Tennessee | HOLLADAY x MANOKIN | 4.7 | H | H | C | 2013 | Unknown |
| R10-197 | University of Arkansas | 5002T x R04-357 | 4.7 | T | C | G | 2013 | Unknown |
| R10-1288 | University of Arkansas | S00-9925-10 x UA 4805 | 4.7 | T | C | C | 2013 | Unknown |
| R09-1822 | University of Arkansas | UA 4805 x R01-4747RR | 4.7 | T | C | C | 2013 | Unknown |
| DS25-1 | USDA Stoneville, MS | DT98-9102 x PI 587982A | 4.8 | T | C | C | 2013 | Unknown |
| R07-10322 | University of Arkansas | R97-1634 x V00-3824 | 2.0/5.0/4.3 | H | H | C | 2011/2012/2013 | Unknown |
| N07-14182 | USDA-ARS, Raleigh, NC | N7002 x Clifford | 5.0 | T | C | C | 2012 | Unknown |
| N08-145 | USDA-ARS, Raleigh, NC | N99-186 x TN99-117 | 5.0/4.3 | H | H | C | 2012/2013 | Unknown |
| N08-391 | USDA-ARS, Raleigh, NC | S99-1171 x N00-370 | 5.0/3.6 | T | C | C | 2012/2013 | Unknown |
| N08-521 | USDA-ARS, Raleigh, NC | G96-2272 x N00-377 | 5.0/4.4 | T | C | G | 2012/2013 | Unknown |
| TN07-754 | University of Tennessee | 5601T × 5002T | 5.0 | T | C | C | 2012 | Unknown |
| R09-209 | University of Arkansas | 5002T x Ozark | 5.0 | T | C | C | 2012 | Unknown |
| R07-1685 | University of Arkansas | 5002T x Ozark | 5.0 | T | C | C | 2012 | Unknown |
| R04-572 | University of Arkansas | MD 4900 x Ozark | 5.0 | T | C | C | 2011 | Unknown |
| V07-5775 | Virginia Tech | V97-2276 x GP26062 | 5.0 | T | C | C | 2012 | Unknown |
| S09-10871 | University of Missouri | S04-10364 x S04-12412 | 5.0/2.7 | T | C | C | 2012/2013 | Unknown |
| N09-13663 | USDA-ARS, Raleigh, NC | N98-7961 x N02-8718 | 5.0/4.3 | T | C | C | 2012/2013 | Unknown |
| N05-7380 | USDA-ARS, Raleigh, NC | N7002 x N98-7265 | 5.0 | T | C | C | 2012 | Unknown |
| NCC09-200719-1-37 | North Carolina State University | Unknown | 5.0 | T | C | C | 2013 | Unknown |
| NCC07-8138 | North Carolina State University | Md 99-6226 x N97-9677 | 4.0/5.0/4.1 | T | C | C | 2011/2012/2013 | Unknown |
| NCC09-135 | North Carolina State University | Unknown | 5.0 | H | H | C | 2013 | Unknown |
| N09-13128 | USDA-ARS, Raleigh, NC | N7002 x TAMAHAKARI-BB | 5.0/3.6 | T | C | C | 2012/2013 | Unknown |
| N09-13189 | USDA-ARS, Raleigh, NC | ROY x PI 5083294 | 5.0 | T | C | C | 2013 | Unknown |
| NCC06-899 | North Carolina State University | R97-1634 x N97-9693 | 2.0/5.0/4.6 | T | C | C | 2011/2012/2013 | Unknown |

†The ratings for reactions to race 3 were as follows: 1 = 0-5 cysts, 2 = 6-10 cysts, 3 = 11-20 cysts, 4 = 21-40 cysts, and 5 = > 40 cysts on the root.

‡ “H” represents heterozygote.
