# Supplementary material for: SNP identification and marker assay development for high-throughput selection of soybean cyst nematode resistance
Source: BMC Genomics. 2015 Apr 18;16(1):314. doi: 10.1186/s12864-015-1531-3 (PMC4407462; doi:10.1186/s12864-015-1531-3)
Supplement: Additional file 2: Table S2. — Primers used for sequencing of GmSHMT and GmSNAP. [file 12864_2015_1531_MOESM2_ESM.docx]

**Adittional file 2: Table S2.** Primers used for sequencing of GmSHMT and GmSNAP

| GmSHMT | GmSHMT2 | GmSHMT3 |  |
| --- | --- | --- | --- |
| F: ACAACACTCTCTCTTCTCGC | F: CAGGCCAAAACTCATAATCTG | F: TAATTTTGGTTGGAGAACAATG |  |
| R: CAGATTATGAGTTTTGGCCTG | R: TGGAGTGCGAATTGGTAACG | R: CTAATCCTTGTACTTCATTTC |  |
|  |  |  |  |
| SNAP1 | SNAP2 | SNAP3 | SNAP4 |
| F: CGCTTATGAATCTTCTTCTTCTTC | F: GGTTTTGGAGTGGGCTGAATC | F: GCAGGAGCAAATTATTTTGCTGTC | F: CACTGTGTAAAGTTAATTTTTTTGCTTAC |
| R: GCATGTAGTGGTAACAACTGAGAC | R: ACCCAGGAGAAGAGATATCAATA | R: GAATTTGATGACACGTACAATAAATG | R: CCAATTCAATTAAAACCAAAGCAGG |
